# Commentary on Singh et al. (2020) Postzygotic Somatic Mutations in the Human Brain Expand the Threshold-Liability Model of Schizophrenia

**DOI:** 10.3389/fpsyt.2021.653624

**Published:** 2021-08-06

**Authors:** Peter S. Spencer, Glen E. Kisby

**Affiliations:** ^1^Department of Neurology, School of Medicine, and Oregon Institute of Occupational Health Sciences, Oregon Health & Science University, Portland, OR, United States; ^2^Department of Basic Medical Sciences, College of Osteopathic Medicine of the Pacific Northwest, Western University of Health Sciences, Lebanon, OR, United States

**Keywords:** schizophrenia, amyotrophic lateral sclerosis, environmental somatic mutagen, brain DNA damage, methylazoxymethanol

## Introduction

Singh and colleagues note that the mammalian brain has a high degree of mosaicism likely caused by postzygotic genetic and epigenetic alterations that may contribute to most multifactorial and complex neurological disorders, for which the authors use schizophrenia to exemplify. They suggest that schizophrenia arises from a sufficient level of inherited, or inherited plus acquired, brain somatic mutations and/or epimutations, a model with particular relevance they suggest to disorders with neurodegeneration and neurodevelopmental manifestation. We wish to extend this model by noting that acquired somatic mutations/epimutations *in the absence of any inherited predisposition* may be sufficient to trigger a neurodegenerative disorder with neurodevelopmental manifestations and links to schizophrenia. Additionally, in accord with the authors' analysis, we note the importance of environmental mutagens as potential triggers of somatic mutation/epimutations that result not only in cancer but also neurodegenerative disease.

## Motor System Degeneration

The neurodegenerative disease of interest here is the Western Pacific Amyotrophic Lateral Sclerosis and Parkinsonism-Dementia Complex (ALS/PDC), which is known principally in residents of and migrants to and from the former disease hot spots of Guam Island (among Chamorros) and Kii Peninsula, Honshu Island (among Japanese) (1). Once thought to have a genetic origin, later a changing environmental component that explained its decline, and subsequently, with the absence of a characteristic genotype and disappearance of the disease on Guam, considered to have a prominent if not exclusive exogenous etiology. Evidence points to the traditional use of cycad seed for food and/or medicine, practices that disappeared in concert with post-WW II modernization. On Guam, cycad seed, and the food derived therefrom, contain methylazoxymethanol (MAM) β-D-glucoside (cycasin) and β-*N*-methylamino-L-alanine (L-BMAA), an uncommon amino acid (2). These substances are both metabolized to the mutagen formaldehyde (1), which from endogenous sources and/or from exogenous exposure is linked to both brain cancer and sporadic ALS (3). Accordingly, MAM, the active form of the major cycad toxin, is a genotoxic agent with both carcinogenic and neurotoxic properties (1). There is a strongly significant correlation between the concentration of cycasin (but not L-BMAA) in cycad flour prepared Chamorro-style and the incidence of ALS and P-D among males and females on Guam (4). Additionally, MAM disrupts retinal and cerebellar development, which respectively anticipate and attend the adult onset of ALS/PDC (5). Most significantly, for present purposes, MAM is widely used experimentally to produce a rodent model of schizophrenia. In female rats, the administration of MAM on gestational day 17 disrupts brain development leading to histological, neurophysiological and behavioral deficits analogous to those of schizophrenia (6, 7). How closely the rodent MAM model reproduces the neuropathological and behavioral features of schizophrenia is unknown.

## Schizophrenia and ALS

While rare cases have linked schizophrenia and Western Pacific ALS/PDC (8, 9), there is growing clinical, epidemiological and biological evidence of an association between ALS and psychotic illness (10), particularly schizophrenia (11). Westphal (12) observed that the paranoid and manic-depressive states of schizophrenia were associated with ALS but considered the neuropsychological and motor system disorders to be unrelated. Wechsler and Davison (13) reported that mental symptoms were due to cortical degenerative changes associated with ALS. Turner and colleagues (14) found that schizophrenia may represent a risk factor for ALS (OR 5.0). Howland (15) noted several cases in which schizophrenia occurred in ALS patients. Register-based nationwide studies show a higher occurrence of schizophrenia up to 1–5 years before and 2–5 years after ALS diagnosis (16). The coexistence of ALS and schizophrenia has been interpreted as indicative of a shared polygenic basis (11), and GWAS studies support a genetic correlation between the two conditions (11, 17). Neuropsychiatric symptoms other than schizophrenia, including obsessive-compulsive disorder, autism, and alcoholism, occur more frequently in first- or second-degree relatives of ALS patients with and without *C9orf72* expanded repeats (18, 19). Disturbances in motor neuron function have been demonstrated in schizophrenia (20–22).

## Molecular Mechanisms

We have discussed elsewhere evidence that MAM experimentally induces early epigenetic changes that coincide with DNA damage and cell-cycle reactivation, evidence for which is seen in the ALS/PDC brain (23–25). MAM disrupts the cell cycle presumably by inducing DNA damage *via* methylation of guanine (i.e., *N*7 methyl and/or *O*^6^-methyl adducts) that inhibits DNA replication during S phase (26) and disrupts neuroepithelial cells undergoing their final mitosis (27). Some of the early changes induced by MAM in somatic cells include nucleoprotein structural alterations, mitotic abnormalities, and induction of polyploidy (28) as well as retinoblastoma (Rb) gene mutations, which lead to the development of intraocular neoplasms (29, 30). Expression of the retinoblastoma gene is also altered in the prefrontal cortex of rats treated developmentally with MAM (31) and in human neuroprogenitor cells (hNPCs) 24 h after acute treatment with the genotoxin (32). L-BMAA also induces cell-cycle dysregulation in embryonic rat striatal neurons (1, 33).

## Discussion

Whether MAM-induced DNA damage and/or epigenetic changes are the initial event(s) that trigger the cell-cycle changes is presently unknown, but it is clear that this genotoxin induces somatic cell changes that are linked with both experimental schizophrenia and neurodegeneration in the form of Western Pacific ALS/PDC. Given the absence of any known genetic susceptibility factor for this prototypical neurodegenerative disease—one that often has subclinical evidence of developmental cerebellar and retinal dysplasia (5)—it is reasonable to propose that exposure to an environmental mutagen/epimutagen alone (notably MAM) is sufficient to trigger the disorders. Given this conclusion, we extend the model proposed by Singh and colleagues to include *environmentally acquired* somatic mutations/epimutations as sufficient to trigger a neurodegenerative disorder with neurodevelopmental manifestations and links to schizophrenia. The corollary emphasizes the need to search for early-life exposure to environmental mutagens/epimutagens in related spontaneous neurodegenerative disorders, including ALS, atypical Parkinson syndromes such as Progressive Supranuclear Palsy, and Alzheimer disease (34).

## Author Contributions

All authors listed have made a substantial, direct and intellectual contribution to the work, and approved it for publication.

## Conflict of Interest

The authors declare that the research was conducted in the absence of any commercial or financial relationships that could be construed as a potential conflict of interest.

## Publisher's Note

All claims expressed in this article are solely those of the authors and do not necessarily represent those of their affiliated organizations, or those of the publisher, the editors and the reviewers. Any product that may be evaluated in this article, or claim that may be made by its manufacturer, is not guaranteed or endorsed by the publisher.
